# Dr. A. P. Elston

**Published:** 1854-11

**Authors:** 


### DR. A. P. ELSTON.

The profession in our city has lost an amiable and accomplished member in the death of Dr. A. P. Elston, which took place in this city, after a lingering illness, a few weeks since. The following proceedings commemorative of the melancholy event were handed us too late for insertion in our last number:

The physicians of Louisville having assembled at the office of Dr. Rudd, on the 6th inst., for the purpose of noticing, in a suitable manner, the recent death of their esteemed associate, Dr. Allen P. Elston; Dr. G. W. Smith was called to the chair, and Dr. Bartlett appointed Secretary; it was

*Resolved*, That we cherish, with fraternal gratification, a remembrance of the worthy ambition and high culture which promised so much in the earlier years of our friend's professional life, and lament that so many of the latter portion of them were rendered useless and disheartening to him in protracted disease.

*Resolved*, That while we commemorate his acknowledged merits as a practitioner of medicine, and his scrupulous observance of the principles which regulate honorable professional intercourse, we cannot forget his remarkable urbanity as a gentleman, and those estimable social qualities that rendered him a favorite in all circles of intelligent and polite society.

*Resolved*, That we sympathize with his family in their bereavement, and that we will unite with them in paying the last tribute of respect to his earthly remains by attending the funeral services.

DR. G. W. SMITH, *Chairman*.

Dr. John Bartlett, *Secretary*.

